# Hepatic resection due to a fish bone

**DOI:** 10.1016/j.ijscr.2021.105722

**Published:** 2021-03-05

**Authors:** Carlos E. Costa Almeida, Teresa Caroço, Marta Silva, José Miguel Baião, Andreia Guimarães, Miguel Ângelo

**Affiliations:** a Faculdade de Medicina da Universidade de Coimbra, Coimbra, Portugal; b Instituto Português de Oncologia de Coimbra Francisco Gentil, Coimbra, Portugal; c Centro Hospitalar e Universitário de Coimbra, Coimbra, Portugal

**Keywords:** Hepatic abscess, Pyogenic, Foreign body, Liver resection

## Abstract

**Introduction and importance:**

Hepatic abscess due to foreign body is rare. Diagnosis is difficult and drainage may not be enough. Hepatic resection may be indicated.

**Case presentation:**

A 76yo male patient resorted to the emergency room because of fever and abdominal pain. Abdominal CT diagnosed a hepatic abscess in the left lobe due to a fishbone. Percutaneous and surgical drainage failed. Laparotomic hepatic resection was necessary. The patient was discharged asymptomatic.

**Clinical discussion:**

Liver abscess due to foreign body usually occur in the left lobe. Drainage and removal of the foreign body are mandatory and can be achieved either by percutaneous or surgical drainage. Resection is rarely indicated but sometimes is the only way. The authors found only ten cases of hepatic resection due to pyogenic abscess because of a foreign body.

**Conclusions:**

Hepatic resection is rarely needed to treat a pyogenic abscess, but surgeons must be aware and able to proceed with it.

## Highlights

• Hepatic abscess due to a foreign body is rare.
• Diagnosis is difficult since symptoms are non-specific.
• Stomach and duodenum are the common sites of perforation.
• Antibiotics with abscess drainage and foreign body removal is the most frequently used treatment.
• Hepatic resection is rarely needed, but surgeons must be aware of its possibility.

## 1 Introduction and importance

Foreign body ingestion is not uncommon, but complications only occur in 1% of cases [[1], [2], [3], [4]]. It was 1898 when Lambert reported the first liver abscess due to a gastrointestinal perforation by a foreign body [[2], [3], [4]]. Although several reports have been published since then, a hepatic abscess because of foreign body ingestion is still a rarity [[2], [3], [4],^5^]. Its diagnosis is not easy because most of the patients do not remember the foreign body ingestion event and usually present with non-specific symptoms [2,3,5]. Removal of the foreign body is crucial and sometimes drainage may not be enough. In this setting, the authors report a rare case of a patient with a pyogenic abscess due to a fishbone who had to be submitted to hepatic resection. This work has been reported in line with SCARE 2020 criteria [6].

## 2 Case presentation

A 76 years-old male patient resorted to the emergency room (ER) because of abdominal pain for the last 72 h, asthenia, anorexia, and fever (39 °C). Neither diarrhea nor constipation was present. No event was associated with the complaints. No jaundice. He had tenderness on the right upper abdominal quadrant, no guarding, Murphy’s sign was absent and peristaltic sounds were normal. No masses. He was hemodynamically normal (130/78 mmHg, 68 bpm). The patient had been diagnosed with a urinary infection the day before and was discharged home medicated with amoxicillin plus clavulanic acid.

**Fig. 1:**
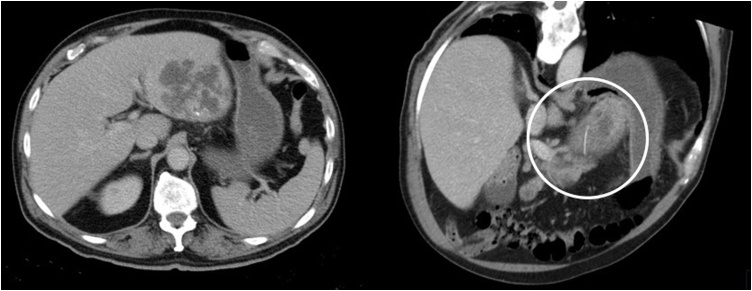
Abdominal CT scan at admission. A multiloculated hepatic abscess in the left lobe is noted with a high-density linear image suspected of being a foreign body.

Blood samples revealed a WBC (white blood count) of 21.3 × 10^3^/uL, no anemia, platelets 220 × 10^3^/uL, PCR (protein C-reactive) 34.9 mg/dL, total bilirubin 33.0 umol/L (<22), conjugated bilirubin 7.7 umol/L (<5.0), alkaline phosphatase of 204 U/L (38–126), AST (aspartate aminotransferase) 415 U/L (15–46), ALT (alanine aminotransferase) 251 U/L (13–69), LDH (lactate dehydrogenase) 965 U/L (313–618). The urine sample excluded infection. Chest film and plain abdominal X-ray were normal. Abdominal ultrasonography (US) revealed a heterogenic area in the left hepatic lobe with 9 cm, with undefined limits. No foreign body was seen. A CT scan showed in the left hepatic lobe an 8.7 cm undefined image, without enhancement, with multiple septa, without air bubbles, contained a linear and dense foreign body with 3.8 cm. A hepatic abscess was the diagnosis Fig. 1.

**Fig. 2:**
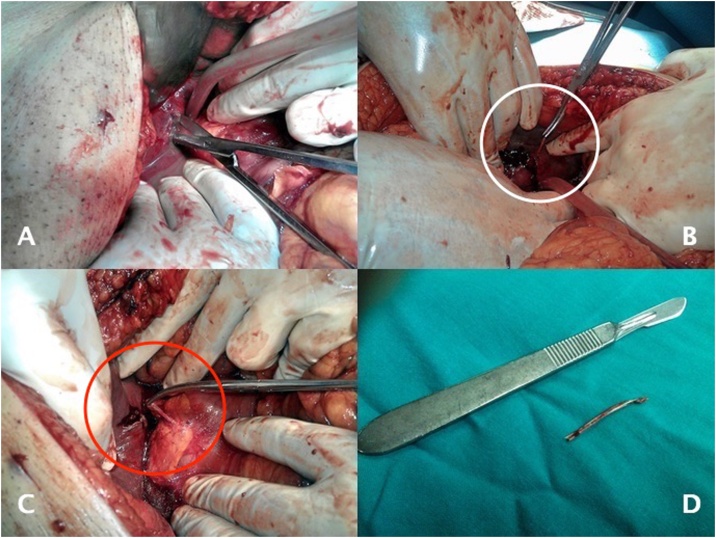
Surgical drainage and fishbone removal. A) Abscess drainage. B) Foreign body removal from inside the liver. C) Fibrous path coming from the pylorus. D) Fishbone with 3.8 cm.

Piperacillin plus tazobactam was initiated. CT-guided percutaneous drainage was conducted collecting greyish pus. Two days later there was scarce drainage, and the patient was not getting better. He was tachycardic, with sustained fever (38.5 °C), abdominal pain, and tenderness. Blood tests revealed a WBC of 26.3 × 10^3^/uL and PCR 33.8 mg/dL. A septic shock was diagnosed, and a midline laparotomy was performed to drain the abscess. An occluded fibrous path from the pylorus to the left lobe was noted as the possible site of foreign body migration following duodenum perforation. The 3.8 cm fishbone was removed from inside the liver. Fig. 2. One drain was placed inside the abscess and another under the liver, and the patient went to the Intensive Care Unit (ICU). Both blood culture and pus microbiology were positive for *Streptococcus constellatus*. Levofloxacin was initiated with the previous antibiotic.

**Fig. 3:**
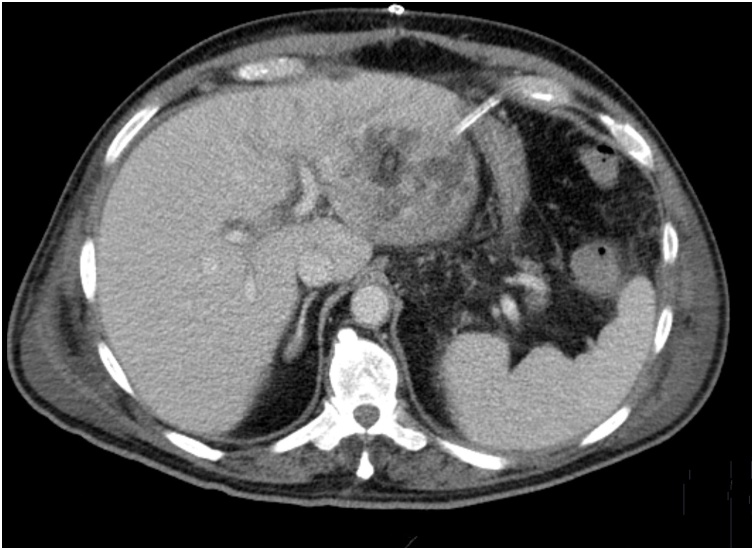
Abdominal CT scan seven days after surgical drainage. An ongoing multiloculated abscess in the left lobe is identified with a drain inside.

On the seventh postoperative day, fever recurred. Drainage was absent for the last 48 h. WBC of 21.6 × 10^3^/uL and PCR 23 mg/dL. An abdominal CT scan was repeated showing an ongoing lobulated image in the left lobe, multiloculated, slightly smaller, with a drain within and moderated peritoneal fluid. Fig. 3. We decided to proceed with an open atypical hepatic resection of segments II and III. The operation was uneventful.

On the fourth postoperative day, the patient was asymptomatic, hemodynamically normal, and without fever. He was transferred to the General Surgery ward on oral diet and normal bowel movements. On postoperative day 12, he was discharged home asymptomatic. At 6 months follow-up patient was asymptomatic.

## 3 Clinical discussion

Liver abscess etiology is variable and localization within the liver may reveal its origin. The biliary system is responsible for 35–40% of pyogenic abscesses, intestinal disease (appendicitis, diverticulitis, malignant colonic perforation) for 20%, contiguous extension (perforated ulcers, gangrenous cholecystitis), trauma (blunt or penetrating) for up to 5%, arterial embolization of bacteria through the hepatic artery (drug abuse, hepatic artery chemoembolization, distant infections of heart, lungs, kidney, bone, teeth) for 12% of cases, cryptogenic abscess for 10–45% [2,7]. A penetrating hepatic trauma following a gastrointestinal perforation by a migrating foreign body is extremely rare [[1], [2], [3]]. Although several reports have been published since the first case report in 1898 by Lambert, there are only 62 cases of hepatic abscess secondary to a fishbone according to a Gonçalves et al. review [1,2,4].

Signs and symptoms are non-specific making diagnosis difficult [[2], [3], [4],^7^,^8^]. The classic triad of fever, jaundice, and right upper abdominal pain is only present in less than 10% of cases [2,4,7]. Most patients present with fever (92%) and abdominal pain (50%). Nausea, vomiting, malaise, chills, cough, anorexia, hepatomegaly, abdominal mass and ascites, can also be present [2,7]. Leukocytosis is present in up to 90% of patients, as well as elevated phosphatase alkaline, bilirubin, and transaminases (50–67%) [7]. In the case presented symptoms were non-specific, the reason why he was misdiagnosed and discharged home the day before. Although the classical triad was not present, clinical presentation and the blood test could have raised the hypothesis of a hepatic abscess.

Abscesses originating from the biliary system or hepatic artery are usually multiple and small, while those caused by trauma (including ingested foreign bodies) or cryptogenic are usually big and unique. The former affects both hepatic lobes (90%), while the remaining usually affects the right lobe [7]. Hepatic abscess due to a foreign body migrating from the gut is extremely rare. Only 1% of ingested foreign bodies cause gastrointestinal perforation [[1], [2], [3], [4]]. Fishbone, toothpick, chicken bone, needle, clothespin, rosemary twig, lobster shell, metal wire, and pen are the foreign bodies implicated. Although perforation can occur at any site of the gastrointestinal tract, the ileocecal junction and rectosigmoid are the most frequent locations. However, the stomach (including pylorus) and duodenum are the common sites of perforation by foreign bodies migrating into the liver [[1], [2], [3], [4],^8^]. In this setting, a hepatic abscess due to a foreign body frequently affects the left lobe [[1], [2], [3]]. Other possible sites are the right colon and the transverse colon but, in these cases, the abscess is frequently found in the right lobe [3]. There can be a long period of time from the ingestion and perforation until the onset of symptoms. Probably the thicker gut wall (stomach) allows for a gradual and slow perforation, while the adjacent omentum and liver can help to seal the perforation avoiding peritoneal spillage of gut contents [3]. Inquiring about foreign body ingestion is paramount. However, most of the patients do not remember such an event, which can lead to a misdiagnosis and treatment delay [[1], [2], [3], [4],^5^,^8^,^9^]. Factors such as alcoholism, psychiatric illness, age extremes, selected professions (ex. carpenters), eating quickly, cold and hot beverages, inmates, cognitive impairment, and use of dentures are associated with a higher risk of foreign bodies ingestion [1,3,5,8]. In the case reported the abscess was in the left lobe, which should have raised the suspicion of a foreign body as the causing agent. The finding of a closed fibrous path between the pylorus and the liver supports the idea of a perforation of the gut by the fishbone and the slow migration into the liver. Perforation was sealed allowing for a late onset of symptoms.

Plain abdominal radiography will not diagnose an abscess and has limited utility in detecting a fishbone, which depends on density that varies between fish species. For instance, codfish and salmon have a high-density skeleton [[1], [2], [3],^5^]. Ultrasonography (US) is a good technique, but an abdominal CT scan is the golden standard to diagnose a hepatic abscess and to detect a foreign body (sensitivity up to 90%) [1,3]. However, a fishbone may get obscured when either oral contrast or intravenous contrast is given. Additionally, perforation site and imaging findings of gut perforation may not be visible [2,3]. Magnetic Resonance Image (MRI) should not be used when a metallic foreign body cannot be excluded [3,4]. In the case reported the US was not conclusive and did not found the foreign body. The CT scan was essential for diagnosing the hepatic abscess and the foreign body. Although the perforation site was not identified in the CT, treatment could only be planned after it. This supports the idea that an early CT scan should be promptly performed [3].

Although there is no consensus on the best treatment for a hepatic abscess caused by a foreign body, systemic antibiotics plus percutaneous/surgical drainage and removal of the foreign body remains the most frequently used approach [1,2,7]. Some authors are advising systemic antibiotics alone for pyogenic abscesses with less than 5 cm, promoting percutaneous drainage only for lesions > 5 cm. Other authors recommend surgical drainage as the best option for abscesses > 5 cm [2]. Lanthaler et al. reported an endoscopic removal of a toothpick perforating the gastric wall into the liver plus repeated endoscopic rinsing of a perigastric abscess, but in the end, resection was necessary [10]. According to the literature, only two cases of hepatic abscess caused by a foreign body were successfully treated with antibiotics alone [2]. One case treated by percutaneous and surgical drainage recurred because the foreign body was not removed [11]. El Asmar et al. reported a case of a hepatic abscess due to a toothpick that grew on antibiotics only (3.3 cm–6.0 cm), ending in a laparotomic wedge resection [12]. Chen et al. reported a resolution rate of only 9,5% when the foreign body is not removed [3]. It seems clear that removal of the foreign body is mandatory to both treat and avoid recurrence [1,2]. Conservative treatment with antibiotics only is accepted as first-line for patients unfit for invasive approaches, but always as a bridge to foreign body removal. Although removal can be achieved by endoscopy (when a part of the foreign body is still in the gut), transluminal endoscopy or US/CT guided percutaneous intervention, surgery still is the best treatment when the diagnosis is highly suspected or certain [3,12]. Open or laparoscopic surgery are both valid options. Laparotomy virtually treats all patients, but since 2011 laparoscopy is increasing. Laparoscopy has the advantage of magnifying the operative field and the light may reflect the foreign body making it easier to be found [3]. In a review conducted by Tomoaki et al., from nine patients only two were submitted to surgery as first-line treatment and only one had resection performed to control the infection [2]. At the time our patient was treated (2012), the surgical team experience in emergency laparoscopic hepatic surgery was minor. Adding to the fact that open surgery virtually treats all patients, the surgical team decided on a laparotomy. At the present day, the authors would probably promote a laparoscopic approach.

Hepatic resection is rarely necessary to treat this potentially fatal condition. In a PubMed research (English written papers) we could only find ten reports of hepatic abscess due to foreign bodies treated by hepatic resection. Our report is the eleventh (Table 1). Laparotomy [[8], [9], [10],^12^,^13^] and laparoscopy [3,4,[14], [15], [16]] were used for hepatic resection in five patients each. Surgery was the first option in only five cases [3,8,9,12,14].

**Table 1:** Published cases (ten) of hepatic abscess due to a foreign body treated by resection. N/A – not available.

	Author, Year	Age, Gender	Foreign body	Perforation site	Abscess location	Size (cm)	First treatment	Surgical approach	Resection type
1	Chen et al. 2019 [3]	37, Male	fishbone	stomach	left lobe	4.5	surgery	laparoscopy	segment III
2	Abu-Wasel et al. 2012 [4]	45, Female	toothpick	N/A	left lobe	4.0	antibiotics	laparoscopy	left lobe
3	Pan et al. 2015 [8]	52, Female	toothpick	stomach	left lobe	N/A	surgery	laparotomy	left lobe
4	Kanazawa et al. 2003 [9]	48, Female	toothpick	antrum	left lobe	N/A	surgery	laparotomy	atypical
5	Lanhaler et al. 2009 [10]	38, Male	toothpick	anterior gastric wall	caudate lobe	6.0	upper endoscopy	laparotomy	segment I
6	El Asmar et al. 2017 [12]	61, Female	toothpick	colon	segment V	3.3	antibiotics	laparotomy	atypical
7	Pederson et al. 1986 [13]	66, Male	toothpick	antrum	left lobe	N/A	surgery	laparotomy	atypical
8	Riani et al. 2012 [14]	68, Male	N/A	N/A	left lobe	N/A	antibiotics	laparoscopy	left lobe
9	Currò et al. 2017 [15]	82, Female	toothpick	N/A	left lobe	8.0	surgery	laparoscopy	left lobe
10	Li et al. 2019 [16]	58, Male	fishbone	stomach	left lobe	N/A	antibiotics	laparoscopy	left lobe
11	Case reported	76, Male	fishbone	pylorus	left lobe	8.7	percutaneous drainage	laparotomy	atypical

In the case reported percutaneous drainage without foreign body removal was the first approach. Laparotomic drainage and removal of the foreign body were then necessary but unable to avoid a septic shock. An emergent laparotomic atypical resection of the left hepatic lobe was conducted to control the infection. In a retrospective analysis and taking into account the multiloculated feature of the large abscess, we probably should have performed surgical drainage (laparoscopic or laparotomic) as a first-line treatment with foreign body removal. Even though, we can not know if early surgical drainage could avoid the hepatic resection. Although surgical drainage is the best option, sometimes it fails, and resection is necessary. This report presents a rare case of failure of the surgical drainage plus foreign body removal, treated with hepatic resection.

## 4 Conclusions

Hepatic abscess due to a foreign body is extremely rare. The stomach and duodenum are the common sites of perforation. Antibiotics plus abscess drainage and foreign body removal is the most frequently used treatment. Hepatic resection is rarely needed, but surgeons must be aware of its possibility and be able to proceed with it.

## Declaration of Competing Interest

The authors report no declarations of interest.

## Sources of funding

No sources of funding.

## Ethical approval

No need for ethical approval since this is not a research paper.

## Consent

Written informed consent was obtained from the patient for publication of this case report and accompanying images. A copy of the written consent is available for review by the Editor-in-Chief of this journal on request.

## Author contribution

CE Costa Almeida: data collection, interpretation, writing paper, review.

M Silva: review.

Jose M Baião: review.

A Guimarães: review.

M Angelo: review.

T Caroco: writing paper, review.

## Registration of research studies

Not applicable.

## Guarantor

Carlos Manuel Costa Almeida, MD, PhD (chairman of Surgery Department).

## Provenance and peer review

Not commissioned, externally peer-reviewed.
